# Mapping Geographic Trends in Early Childhood Social, Emotional, and Behavioural Difficulties in Glasgow: 2010–2017

**DOI:** 10.3390/ijerph191811520

**Published:** 2022-09-13

**Authors:** Samantha Ofili, Lucy Thompson, Philip Wilson, Louise Marryat, Graham Connelly, Marion Henderson, Sarah J. E. Barry

**Affiliations:** 1 Department of Mathematics and Statistics, University of Strathclyde, Glasgow G1 1XQ, UK; 2 Centre for Rural Health, Centre for Health Science, University of Aberdeen, Inverness IV2 3JH, UK; 3 School of Health Sciences, University of Dundee, Dundee DD1 4HJ, UK; 4 School of Social Work and Social Policy, University of Strathclyde, Glasgow G4 0LT, UK

**Keywords:** child development, disease mapping, multilevel modelling, SDQ, spatiotemporal, children, social and emotional difficulties, early years

## Abstract

Measuring variation in childhood mental health supports the development of local early intervention strategies. The methodological approach used to investigate mental health trends (often determined by the availability of individual level data) can affect decision making. We apply two approaches to identify geographic trends in childhood social, emotional, and behavioural difficulties using the Strengths and Difficulties Questionnaire (SDQ). SDQ forms were analysed for 35,171 children aged 4–6 years old across 180 preschools in Glasgow, UK, between 2010 and 2017 as part of routine monitoring. The number of children in each electoral ward and year with a high SDQ total difficulties score (≥15), indicating a high risk of psychopathology, was modelled using a disease mapping model. The total difficulties score for an individual child nested in their preschool and electoral ward was modelled using a multilevel model. For each approach, linear time trends and unstructured spatial random effects were estimated. The disease mapping model estimated a yearly rise in the relative rate (RR) of high scores of 1.5–5.0%. The multilevel model estimated an RR increase of 0.3–1.2% in average total scores across the years, with higher variation between preschools than between electoral wards. Rising temporal trends may indicate worsening social, emotional, and behavioural difficulties over time, with a faster rate for the proportion with high scores than for the average total scores. Preschool and ward variation, although minimal, highlight potential priority areas for local service provision. Both methodological approaches have utility in estimating and predicting children’s difficulties and local areas requiring greater intervention.

## 1. Introduction

It is estimated that one in every five children under 7 years old meets the criteria for a mental health diagnosis [1]. Identifying mental health problems when they first become evident in younger children [2] and delivering early interventions can reduce the individual and societal impact [3]. We can assess the presence of social, emotional, and behavioural difficulties (e.g., rating a child’s willingness to help others, their relationships with peers, emotional regulation, and inattention), which indicate if a child is at risk of mental health problems [4,5]. Childhood social, emotional, and behavioural problems can predict school exclusion [6] and later mental health conditions [7] at an individual level, and lead to higher healthcare and social welfare costs at a societal level [8,9,10]. Identifying patterns and trends in early childhood mental health can support the development of targeted interventions.

This paper focuses on geographic patterns in mental health according to where a child lives. Geographic variation in outcomes can be expected as the residential environment influences the day-to-day social and physical experiences of the child and their caregiver. There has been considerable research on how the characteristics of a neighbourhood are associated with child mental health and wellbeing [11,12,13], with deprivation being the most commonly researched factor. Considerable differences in outcomes may point to health inequalities that would benefit from intervention.

There is no common theoretical or methodological framework to investigate geographic differences in child mental health [13] or the effectiveness of place-based interventions [14]. Ebener et al. present a hierarchy of geospatial techniques that support decision making in early childhood [15]: mapping, spatial analysis, and spatial modelling. This process is facilitated by population data, giving an adequate sample size to provide small area estimates (e.g., [16,17]). Visualising differences between areas through mapping can highlight areas in need of further action. In Australia, mapping the proportion of children at risk of mental disorders (according to their profile of conduct behaviours) in 2009 showed how the risk was greater in rural areas of high deprivation [18]. Relationships between neighbouring areas can be quantified—whether they are more similar (demonstrating spatial correlation) or dissimilar (spatial heterogeneity)—to determine if resources should be spatially targeted or universal. For example, in the United States, children living nearby to each other were found to have similar levels of behavioural problems, ref. [19] supporting the use of place-based approaches. Spatial modelling estimates the spatial pattern of risk, the influence of covariates on risk, and improves small area estimates compared with summarising the observed data alone [20]. In both the United Kingdom (2010–2012) [21] and in Canada (2005–2007) [22], spatial models showed that variation in the risk of mental health difficulties and of behavioural problems, respectively, which remained after controlling for demographics—suggesting that other factors may contribute to differences. In both cases, it was not determined whether these reflected stable, long-term differences.

There is increasing literature on the importance of considering time in spatial research [23]. The composition (demographics of the individuals in the neighbourhood) and characteristics of neighbourhoods change over time, affecting the overall spatial pattern. Spatial modelling can be easily extended to consider the effect of time forming spatiotemporal models [24]. Such models estimate the main spatial pattern that is consistent across the time, the temporal trend that is common to all areas, and/or the spatiotemporal interactions that describe how an area changes over time.

Spatial and spatiotemporal techniques typically use aggregated outcomes, e.g., an average or total for an area. Disease mapping [25], a commonly used form of spatial modelling, maps the overall risk of health outcomes in a region to show small areas that deviate from the region overall and how the relationship between areas is explained by covariates (e.g., [20]). Aggregating the outcome by small area is useful as it can be easily modelled to support local level action. By focusing on an aggregated outcome, however, this approach is limited by ecological fallacy, and we are only able to make inferences about the level at which the outcome is derived (i.e., the area), and thus cannot make conclusions about the individual. An alternative approach is a multilevel [26] spatial approach, in which the outcome of interest is at an individual level and the variation between areas can be estimated (e.g., [21,22]). In this case, information is retained about each child and their neighbourhood, avoiding loss of information. Considering individual, spatial, and spatiotemporal effects adds further complexity to the model [27].

The approach chosen to evaluate child mental health trends can influence decision-making. We are interested in looking at the impact of methodology in identifying geographic variation in early child mental health in the United Kingdom. The current study aims to apply disease mapping and multilevel approaches to do the following:

1. Map the overall risk of preschool children’s social and emotional difficulties in an entire city from 2010–2017;
2. Analyse the relationship between preschool children’s social, emotional, and behavioural difficulties in neighbouring areas across a whole city from 2010–2017;
3. Investigate the extent to which the relationship between geography and preschool children’s social, emotional, and behavioural difficulties is explained by demographics;
4. Explore the temporal and spatiotemporal trends in preschool children’s social, emotional, and behavioural difficulties across an entire city from 2010–2017.

## 2. Methods

### 2.1. Setting

The ChiME (Child Mental Health in Education) study took place in Glasgow City, Scotland, from 2010–2017, the most populated city in Scotland, United Kingdom. It has been shown that the levels of social, emotional, and behavioural difficulties in Glasgow are similar to the rest of the UK at preschool age [28].

### 2.2. Study Design

The ChiME data were routinely collected by the Glasgow City Council, providing a whole population sample from 2010–2017 (derived from the Triple P parenting intervention [29]). Previous spatial analysis using a subset of ChiME data is published elsewhere [21].

Preschool staff were asked to complete the teacher version of the Strengths and Difficulties Questionnaire (SDQ) [30]. Forms were completed on paper (2010), through a mix of paper and electronic submissions (2011) or solely electronically (2012–2017) using the education services’ information management system. For 2010 and 2011, the SDQ version used was for 4–16-year-olds. This was changed to the version for 2–4-year-olds in subsequent years due to staff perception that question wording for conduct problems in that version was more developmentally appropriate.

All children living in Glasgow, who were attending a local authority preschool or private preschool with funded places in 2010–2017, and who were due to start school the following year, were eligible. During the study period, there were 99,346 children aged 4–5 years in Glasgow [31]. In total, data were collected from 41,128 children from 182 preschools in the study. Children in private nurseries without funded places or those not in preschool were not part of the study. Approximately 90% of 4-year-old children in Scotland are registered in some form of early learning and childcare. There is no relationship between being registered in a preschool and area deprivation in Scotland [32].

### 2.3. Variables

Biological sex at birth and date of birth were obtained from educational services’ administrative databases and were linked to the ChiME database. Age was calculated in years from planned start in primary school (1 August following data collection). Each child was assigned a Scottish Index of Multiple Deprivation (SIMD) [33] score based on their residential postcode and cohort. As a result of the local authority’s considerable levels of socioeconomic deprivation, scores were categorised into quintiles within Glasgow rather than for the whole of Scotland to form a local index. In the absence of household data, deprivation quintiles were used as a proxy for household socioeconomic status.

### 2.4. Outcome

In the SDQ, there are 25 questionnaire items scored on a three-point Likert scale (0, 1, 2) across five domains: emotional symptoms, conduct problems, hyperactivity/inattention, peer problems, and prosocial behaviour. The first four of these domains contribute to a “total difficulties” score out of 40. Scoring was based on the code provided on the SDQ website [34]. New banding classifies scores from 0–10 as close to average, 11–14 as slightly raised, 15–17 as high, and 18 and above as very high for 2–4-year-olds in the UK [35]. High scores indicate a high likelihood of psychiatric diagnosis.

### 2.5. Geographical Data

Residential postcodes were situated within the 21 electoral wards of Glasgow at that time. Electoral wards were aligned with local planning and policy making and were updated to reflect population change. Two new ward boundaries were introduced after the study period in 2017, creating 23 boundaries, with a new ward created in both the east and west of the city. The ward boundaries were obtained from SASPAC (Small Area Statistics PACkage) at https://saspac.org/ (accessed on 2 July 2020), using the boundaries at 2011.

### 2.6. Statistical Methods

Descriptive analyses compared the median total difficulties scores and the proportion of children with high scores by demographic characteristics, wards, and years.

Models were estimated using Bayesian inference [36], which is increasingly applied in child psychology research to support the analysis of complex data structures [37]. Analysis was conducted in R version 4.0.1 [38] and using package R-INLA (www.r-inla.org, accessed on 2 February 2020) [39] with weakly informative priors [40].

Both models followed the spatiotemporal formulation by Bernardinelli et al. [41]. According to this formulation, we included a linear time trend common to all wards in the city, an overall spatial random intercept (describing how wards deviate from the city-wide average), and a differential time trend (random slope) estimating how much a ward deviates from the linear trend. The intercept and slope may be positively correlated, meaning wards with high difficulties have a higher differential trend, or negatively correlated, where wards with high difficulties have a lower differential trend.

Spatial correlation of residuals aggregated by ward can be measured through Moran’s I statistic [42]. For the multilevel model, we generated residuals by simulating values from the distribution and scaled them using R package DHARMa [43]. The value of I estimated the correlation between adjacent wards—using queen adjacency (wards sharing a border or point were considered neighbouring). The value of I indicates where neighbouring wards were similar (I ≈ 1), dissimilar (I ≈ −1), or had no association (I ≈ 0). Moran’s I test assessed the null hypothesis that the value of I that was observed was equal to zero and dictated the structure of the spatial random intercept in the model.

#### 2.6.1. Disease Mapping Model

The first outcome of interest was the number of children with or at high risk of psychopathology in an area. The outcome was defined as the number of children in each ward and year with a high total difficulties score (≥15). We assumed a Poisson distribution for the outcome, as high scores are discrete and relatively rare.

The model was specified as follows:

(1) $$Y_{j}\;\sim \;Pois\left(E_{j}\theta_{j}\right)\;\theta_{j}=\exp\left(\;\beta_{0}+(\;\beta_{1}+u_{1j})\mathrm{Cohort}+u_{0j}+\beta_{k}x_{j}\right)\;u_{0j}\;\sim \;N\left(0,\;\sigma_{u0}^{2}\right)\;\mathrm{where}\frac{1}{\sigma_{u0}^{2}}\sim {\;\Gamma }^{-1}\left(1,\;0.0005\right);\;u_{1j}\;\sim \;N\left(0,\;\sigma_{u1}^{2}\right)\;\mathrm{where}\;\frac{1}{\sigma_{u1}^{2}}\;\sim {\;\Gamma }^{-1}\left(1,\;0.0005\right).$$

where $Y_{j}$ was the number of high scoring children in ward *j* = 1,…, 21, $E_{j}$ was the population of the ward (used as an offset), and $\theta_{j}$ was the probability of having a high score. In the model, $\;\beta_{0}\;$ was the intercept, $\;\beta_{1}$ was the main linear trend across the eight yearly cohorts, and $u_{0j}$ the ward-level spatial intercept. There was no evidence of a spatial correlation in the model; therefore, the spatial effect was unstructured to model spatial heterogeneity. The random slope $u_{1j}\;$ allowed for separating linear trends for each ward. Although shown here to be independent of random intercepts for simplicity, correlated intercepts and slopes were assessed during model building. Coefficient $\beta_{k}$ was associated with ward characteristic *k.* Ward characteristics were the proportion of children outside the expected age for school start (under 4.5 years or over 5.5 years), the proportion of children in the most deprived quintile, and the proportion of boys. The parameters were added to the model in the order described and were retained based on the Deviance Information Criterion (DIC) [44], with lower values indicating better fit.

#### 2.6.2. Multilevel Model

The outcome of interest was the total difficulties score for an individual child, which was modelled using a multilevel model. An advantage of using this outcome is that we can examine how scores change on average, rather than solely focusing on whether a child has reached a cut-off. This still has clinical relevance as population average scores relate to rising rates of psychiatric diagnosis [45]. Furthermore, by retaining the individual level data, a multilevel approach that considers multiple contextual effects can be applied.

Normative SDQ data of children in the UK show that individual scores are skewed, with many zeros and large variance requiring a distribution that can model zero inflation and overdispersion, respectively [46]. Therefore, we assumed a zero-inflated negative binomial distribution [47,48]. The mean of the model is λ, p is the zero-inflation parameter, and *r* is the overdispersion parameter.

(2) $$Y_{ijk}\;\sim \;NB\left({\;\lambda }_{\mathrm{ijk}},p,r\;\right)\;\begin{array}{c} {\;\lambda }_{\mathrm{ijk}}=exp\left(\;\beta_{0}+(\;\beta_{1}+u_{1j})\mathrm{Cohort}+\alpha_{k}+v_{0j}+\beta_{\varphi }x_{i}\right)\; \end{array}\;\alpha_{k}\sim \left(0,\sigma_{\alpha }^{2}\right)\;\mathrm{where}\;\frac{1}{\sigma_{a0}^{2}}\;\sim {\;\Gamma }^{-1}\left(1,\;0.0005\right);\;v_{0j}\sim \left(0,\sigma_{v0}^{2}\right)\;\mathrm{where}\;\frac{1}{\sigma_{v0}^{2}}\;\sim {\;\Gamma }^{-1}\left(1,\;0.0005\right).$$

Here, child *i* = 1,…, N was nested within ward *j* = 1,…, 21 and preschools *k* = 1,…, 180. In the model, $\;\beta_{0}$ was the intercept, $\beta_{1}$ the coefficient for the overall time trend, $v_{0j}$ the ward-level spatial intercept, and $\alpha_{k}$ a preschool effect. There was no evidence of a residual spatial correlation of ward level residuals in the model; therefore, the spatial effect was unstructured. The preschool effect and ward were cross-classified (i.e., they were not hierarchically nested). Random slope $v_{1j}$ allowed for separating linear trends for each ward. As in the disease mapping model, correlated random intercepts and slopes were considered. Coefficient $\beta_{\varphi }\;$ was associated with individual-level variables φ—age (centred on mean age of 59 months and squared), sex, and deprivation quintile. The parameters were added to the model in the order described and were retained based on the Deviance Information Criterion (DIC) [44].

#### 2.6.3. Inference

For both modelling approaches, relative rates (RR) were derived from exponentiated parameters. For example, RR = 1.10 would mean there was a 10% higher rate or score, while RR = 0.90 would mean it was 10% lower. In the disease mapping approach, RR > 1 represented an increase in the number of high scoring children relative to the population of the ward. In the multilevel approach, RR > 1 indicated an increase in the scores of individual children, on average.

The exponentiated intercepts for the ward effects ($\exp\left(u_{0j}\right)\;$and $\exp\left(v_{0j}\right)$ in Equations (1) and (2), respectively, and school effects ($\exp\left(a_{k}\right)$ in Equation (2)) represent the RR for each ward and school compared with the average. The RR of the random slopes ($\exp\left(u_{1j}\right)$ and $\exp\left(v_{1j}\right)$) in Equations (1) and (2), respectively, described how much steeper the linear time trend was in each ward compared with the overall time trend.

Once the covariates were added to the models, the random effects described the unexplained effects after adjustment. Uncertainty in the ward level RR was represented using the exceedance probability, i.e., the probability that the corresponding RR was greater than 1. Exceedance probability values of 0.8 or above were considered to indicate high certainty in the elevated RR.

## 3. Results

### 3.1. Descriptive Data

Of the 41,128 in the initial dataset, children were excluded if they lived outside Glasgow, were missing date of birth, were under 4 or over 6 years old at primary school entry, had a missing or invalid postcode, or were missing a total difficulties score (n = 5957), resulting in 35,171 included children in 180 preschools. Of the included preschools, 60 returned data every year, while the others were involved for a subset of the study years. The characteristics of the preschools by the number of years they were involved in the study is shown in Supplementary Table S1.

Table 1 shows the distribution of demographic characteristics in the included sample and among those with high scores (SDQ ≥ 15; representing 9.0% of the total sample). Children who were outside the expected age range (4.5–5.5), those living in more deprived areas, and boys were observed to have higher levels of difficulties. The proportion of children with high scores increased over time. The distribution of demographics against total scores and for each year are displayed in Supplementary Figure S1 and Supplementary Table S2.

**Figure 1:**
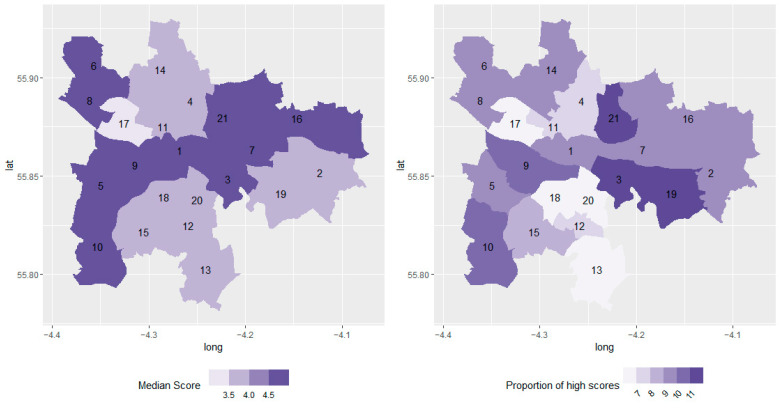
Median and high scores by electoral ward in Glasgow. Median scores (**left**) and proportion of children with high scores (**right**) from 2010–2017. 1. Anderston/City, 2. Baillieston, 3. Calton, 4. Canal, 5. Craigton, 6. Drumchapel/Anniesland, 7. East Centre, 8. Garscadden/Scotstounhill, 9. Govan, 10. Greater Pollok, 11. Hillhead, 12. Langside, 13. Linn, 14. Maryhill/Kelvin, 15. Newlands/Auldburn, 16. North East, 17. Partick West, 18. Pollokshields, 19. Shettleston, 20. Southside Central, 21. Springburn.

In wards 3 and 6, over half of the children were in the most deprived quintile (Supplementary Table S3). While in wards 11 and 17, just under half of children were in the least deprived quintile (Supplementary Table S3). The median score per ward had a narrow range (3 to 5) and there was some geographical clustering of similar median scores (Figure 1, left). The proportion of children with high scores ranged between wards from 6.37% to 11.9% (Figure 1, right). The three areas with the highest rate of high scores were found in the east of the city. Wards 3, 19, and 21 had rates of 11.4%, 11.2%, and 11.9%, respectively. There were several areas in the south of the city (wards 13, 18, and 20) where under 7% of the children had high scores. For outcomes by ward and cohort, see Supplementary Figures S2 and S3.

### 3.2. Disease Mapping Model Results

Table 2 shows the results from the unadjusted and adjusted final disease mapping models. Random slopes were removed from the model as they did not improve fit (DIC = 1023 vs. DIC = 1022) compared with the model without random slopes. For each model, an increase in the relative rate (RR) of high scores per cohort was identified, with an estimated yearly rise of between 1.5% and 5.0% after adjustment. Of the demographics, only the proportion of boys in a ward was associated with the RR of high scoring children in each ward. Although the credible intervals for the other covariates include the null value of one, their inclusion influences the ward variance and removing them did not improve model fit, so they were retained in the model.

**Figure 2:**
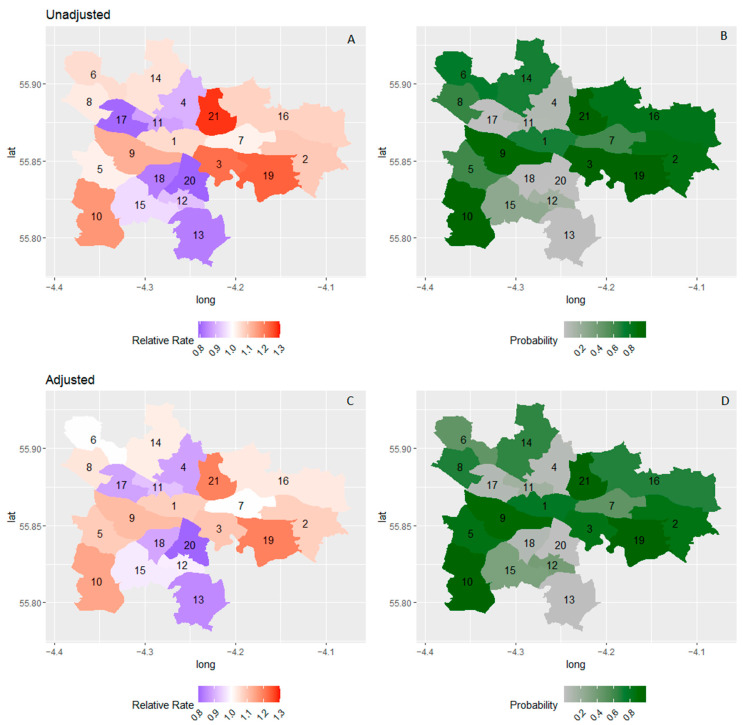
Relative rate increase in number of children with high total SDQ scores before (**A**) and after (**C**) and adjustment and exceedance probability before (**B**) and after (**D**) and adjustment for sex, age, cohort, and deprivation. 1. Anderston/City, 2. Baillieston, 3. Calton, 4. Canal, 5. Craigton, 6. Drumchapel/Anniesland, 7. East Centre, 8. Garscadden/Scotstounhill, 9. Govan, 10. Greater Pollok, 11. Hillhead, 12. Langside, 13. Linn, 14. Maryhill/Kelvin, 15. Newlands/Auldburn, 16. North East, 17. Partick West, 18. Pollokshields, 19. Shettleston, 20. Southside Central, 21. Springburn.

With adjustment for covariates, the mean ward variance fell from 0.026 to 0.018, suggesting that some of the heterogeneity at ward level was due to the demographic composition of the wards. Spatial heterogeneity is demonstrated by the spatial plots in Figure 2. This is most pronounced in ward 3, where RR fell from 21.6% to 9.2%. After adjustment, there was still high certainty (>80%) of increased RR in the south-east (wards 5, 9, and 10) and in the east (wards 2, 3, 19, and 21). With the exception of ward 5, these were mostly wards associated with moderate to high levels of deprivation.

### 3.3. Multilevel Model Results

Table 3 shows the results from the unadjusted and adjusted final multilevel model. The preschool effect improved model fit (DIC = 197,533) compared with the model with only a ward effect (DIC = 198,974). There was no evidence of an interaction between wards and preschools. The random slope did not further improve model fit (DIC = 197,527) and thus was excluded. The average total difficulties score was considerably higher for boys compared with girls by 34.4–39.6%. For every squared monthly deviation in age from the mean of 59 months, RR increased by 0.3–0.4%. Compared with those in the least deprived group, the average total difficulties score consistently increased with rising deprivation. The largest increase was found for the most deprived group, with an average score of 19.3–29.3% higher than the least deprived. There were no interactions between covariates. The credible intervals for the temporal trend showed a 0.3–1.2% increase in total difficulties on average across the years.

**Figure 3:**
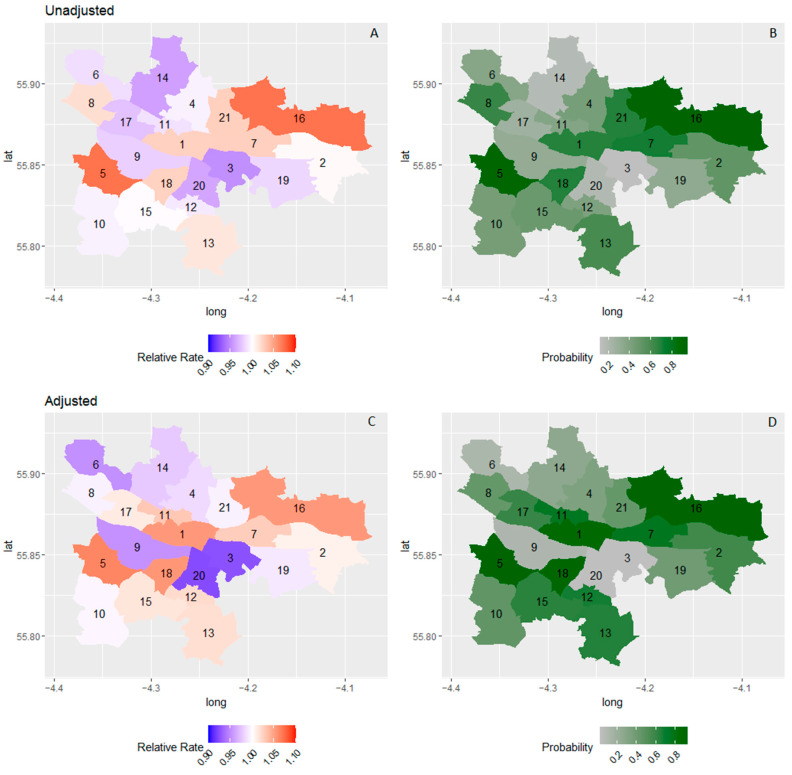
Relative rate increase in average total difficulties scores before (**A**) and after (**C**) and adjustment and exceedance probability before (**B**) and after (**D**) and adjustment for sex, age, cohort, and deprivation. 1. Anderston/City, 2. Baillieston, 3. Calton, 4. Canal, 5. Craigton, 6. Drumchapel/Anniesland, 7. East Centre, 8. Garscadden/Scotstounhill, 9. Govan, 10. Greater Pollok, 11. Hillhead, 12. Langside, 13. Linn, 14. Maryhill/Kelvin, 15. Newlands/Auldburn, 16. North East, 17. Partick West, 18. Pollokshields, 19. Shettleston, 20. Southside Central, 21. Springburn.

There was more variation between preschools than between wards. Adding covariates to the model had a marginal effect on variance estimates. Figure 3 shows the RR associated with living in each ward before and after adjustment for covariates. Before adjustment for covariates, only two wards had a RR greater than 1 with a high certainty (>80%) in the north-east and south-west (wards 5 and 16). After adjustment, there was at least 80% probability that the RR was greater than one for four wards in the centre, south, south-west, and north-east of the city (wards 1, 5, 16, and 18).

### 3.4. Sensitivity Analyses

Because of the changing characteristics of included preschools over the years of the study (shown in Supplementary Table S1), we conducted a sensitivity analysis to investigate whether the temporal trend held for the nurseries that took part in the study every year. Among these preschools, there was still an increasing linear trend among in the disease mapping (RR = 1.054 (1.032–1.076)) and multilevel model (RR = 1.0014 (1.020–1.009)).

Furthermore, we investigated the impact of the choice of disease mapping outcome on the model. The disease mapping approach was repeated using the median difficulties per ward as the outcome. The variation between wards was estimated at zero (0.000 (0.000–0.074)), meaning there was no heterogeneity in the median difficulties per ward.

## 4. Discussion

Our study aimed to identify, map, and model geographic trends in social, emotional, and behavioural difficulty scores of the SDQ in early childhood using differing methodological approaches. A key finding was an overall increase in the number of high scoring children (i.e., children with likely mental health difficulties) in each ward over time from the disease mapping model. We also identified a citywide increase over time in the child score, on average, through the multilevel modelling approach. The rate of increase in the average score was lower than that of the proportion of high scores.

Existing evidence of decreasing temporal trends in teacher-rated SDQ scores for UK children is largely outside this age group, limiting comparability [49,50]. Decreasing trends in parent-rated SDQ in 4–12-year-old girls were found in Scotland from 1995–2014 [51]. The contrasting results in this study may point to differences between teacher-raters and parent-raters, our focus on sub-national outcomes for a whole population, or other factors outside the scope of this study. Other factors that might be relevant include increasing numbers of children entering early years’ provision in Glasgow between 2010 and 2014 [32], changes in approaches to the management of council-run nurseries, and implementation of the Scottish Government’s Early Years Framework, which began around 2010 [52]. Internationally, there have been varying trends in emotional and conduct problems in young children that may reflect cross-cultural or methodological differences [53].

The geographic pattern differed between the approaches. For example, ward 18 (which has low levels of deprivation) was associated with increased average SDQ score in the multilevel model and a low rate of high scoring children in the disease mapping model. The opposite effect was found in ward 9, where there were high levels of deprivation. A single ward was identified with certainty in both modelling approaches as having a level of difficulties in excess of the city as a whole (ward 5, where most children are in the middle quintile for deprivation). There may be unmeasured covariates that would help explain this pattern [54].

Both approaches found minimal variation between wards, as shown by the small variance terms. It could be that wards were not granular enough to capture neighbourhood variability. Despite this, a small number of wards were found to be up to 30% above average for high scoring children and up to 10% higher for average scores. There was no evidence of spatial correlation between neighbouring wards in either model. This implies that an approach focusing on clustered regions would not be appropriate in this case, as discussed by Peel et al. for Canberra, Australia [19,55]. We did not find evidence to support the use of random slopes, meaning the linear temporal trends were consistent across all wards in the city. However, in the multilevel model, wards where children had increased scores on average differed from the geographic pattern of the 2010–2012 subset of the data [21]. It may be that covering a longer time period provided a more robust estimate in this paper.

The multilevel model approach meant the role of preschool and ward could be included. Preschool variance was larger than ward variance, reflecting variation in the measurement of SDQ between the preschools and the need for whole-preschool approaches to support the children in greatest need ahead of school start. This further supports the importance of preschool context in understanding the variation in child mental health outcomes and developing place-based interventions that are informed by preschool-level outcomes, or delivery interventions via preschools [56].

A limitation of the disease mapping model is the fact that characteristics were aggregated by ward. Defining the variables in this way is likely to dilute any existing variations, giving less power to find an effect. In line with prior knowledge, biological sex was identified as important in both approaches. This may reflect differences in how teachers perceive social, emotional, and behavioural problems in young girls and boys [57]. The multilevel model found an association with age and socioeconomic deprivation. This demographic trend is consistent with other settings [21,22,58]. Of the residual variation observed, there were some wards with high deprivation (e.g., wards 20) that were better than expected. This may be partly due to an effect described by Kershaw et al. [59] in Canada as ”off-diagonal” places, where there is a buffering process at the neighbourhood level that reduces the effect of deprivation on development. Conversely, wards 1 and 5 had higher RRs than expected considering their low levels of deprivation (under 10% of children in these wards are in the most deprived quintile). Off-diagonal effects point to the potential presence of other neighbourhood level factors that contribute to spatial variation.

Although the outcomes in each approach are not directly comparable, they both relate to clinical disorder rates at a population [45]. There may be different mechanisms at play for average scoring children compared with those in the high score category, which likely contributed to the difference between trends in our approaches [60].

While there are several population-level child health surveillance programmes in the UK, the outcomes collected [61] and coverage can vary—with children living in the most deprived areas being the least likely to take part [62]. The ChiME study is a unique resource in its ability to address questions about long-term geographic variation in population mental health in young children, in a UK city. This allows the results of this study to be placed into context with international population studies, contributing to our understanding of spatial patterns in child mental health and the role of demographics.

## 5. Conclusions

Our interpretation of the results is that, on average, children’s scores have increased marginally over time, while, at the same time and partly as a result, more children are reaching the high score cut-off. Both effects are consistent across electoral wards, supporting a citywide intervention. The variation between wards can nevertheless be used to identify a small number of priority areas.

The current approach to policy, such as the Scottish National Performance Framework, focuses on excessive rates of high scores [63]. According to the framework, if parent-rated scores increase by more than 1% for 3 years, performance is worsening at a local level. An alternative explanation may be that there is increased reporting of mental health problems [53]. Using the Scottish Government’s guideline, the current study found a worsening performance in the number of high scoring children across 8 years, prompting the need for further action, especially (but not exclusively) in neighbourhoods with high socioeconomic deprivation. Off-diagonal effects show, however, that focusing entirely on deprivation may not always lead to effective place-based interventions [14], which should be directed towards children with the greatest difficulties and should be delivered at preschool-level.

We argue that multilevel modelling of individual scores may be beneficial in identifying priority preschools and areas, warranting further investigation as rising scores transition over the high score cut-off. Therefore, using both approaches together gives deeper insight into the trends in early childhood mental health, which are potentially useful in supporting decision-making.

## Figures and Tables

**Table 1:** Baseline characteristics.

Demographics		N	High Score (%)	Median Score (IQR)
Total		35,171	3149 (9.0%)	
Age (years)	4–4.5	2112	212 (10.0%)	6 (2–10)
	4.5–5	16,456	1541 (9.4%)	5 (2–9)
	5–5.5	15,297	1160 (7.6%)	4 (1–8)
	5.5–6	1306	236 (18.1%)	6 (2–12)
Sex	Female	17,210	877 (5.1%)	3 (1–7)
	Male	17,961	2272 (12.6%)	6 (2–10)
Deprivation Quintile	5 (least deprived)	5071	289 (5.7%)	3 (0–7)
	4	5610	424 (7.6%)	4 (1–8)
	3	6696	605 (9.0%)	4 (1–9)
	2	8179	808 (9.9%)	5 (2–9)
	1 (most deprived)	9615	1023 (10.6%)	5 (2–10)
Cohort	2010	3082	232 (7.5%)	4 (1–9)
	2011	3336	299 (9.0%)	5 (2–9)
	2012	3882	348 (9.0%)	5 (2–9)
	2013	3899	327 (8.4%)	4 (1–8)
	2014	5275	459 (8.7%)	4 (1–9)
	2015	5246	473 (9.0%)	4 (1–9)
	2016	5480	534 (9.7%)	5 (2–9)
	2017	4971	477 (9.6%)	4 (1–9)

SDQ—Strengths and Difficulties Questionnaire, IQR—Interquartile Range.

**Table 2:** Disease mapping model.

	Unadjusted (95% CrI)	Adjusted (95% CrI)
Intercept	0.077 (0.069–0.087)	0.033 (0.017–0.066)
Proportion > 5.5 AND <4.5 years	-	1.467 (0.500–4.283)
Proportion boys	-	3.462 (1.157–10.308)
Proportion most deprived	-	1.326 (0.934–1.839)
Cohort	1.026 (1.009–1.043)	1.033 (1.015–1.050)
$\mathrm{Ward}\;\mathrm{Variance}\;(\sigma_{u0}^{2})$	0.026 (0.011–0.054)	0.018 (0.006–0.041)
DIC	1022	1025

CrI—credible interval; DIC—deviance information criterion.

**Table 3:** Multilevel model.

Title 1	Unadjusted (95% CrI)	Adjusted (95% CrI)
Intercept	5.963 (5.647–6.297)	4.079 (3.886–4.350)
Boys vs. Girls	-	1.370 (1.344–1.396)
Age (centred and squared)	-	1.003 (1.003–1.004)
2nd least deprived vs. least	-	1.115 (1.073–1.159)
middle deprived vs. least	-	1.174 (1.128–1.222)
2nd most deprived vs. least	-	1.234 (1.186–1.284)
Most deprived vs. least	-	1.243 (1.193–1.293)
Cohort	1.007 (1.002–1.011)	1.008 (1.003–1.012)
$\mathrm{Preschool}\;\mathrm{Variance}\;(\sigma_{a}^{2})$	0.071 (0.054–0.092)	0.062 (0.045–0.082)
$\mathrm{Ward}\;\mathrm{Variance}\;(\sigma_{v0}^{2})$	0.011(0.004–0.021)	0.013 (0.008–0.020)
DIC	197,533	196,273

CrI—credible interval; DIC—deviance information criterion.

## Data Availability

Data are owned by Glasgow City Council Education Services and are not currently publicly available.

## Supplementary Materials

The following are available online at https://www.mdpi.com/article/10.3390/ijerph191811520/s1. Figure S1: SDQ outcomes by covariates. Figure S2: Median SDQ scores by Electoral Ward and Cohort. Figure S3: Proportion of children with high SDQ scores by Electoral Ward and Cohort. Table S1: Preschool Demographics. Table S2: Demographics by Cohort. Table S3: Demographics by Ward.
