# Supplementary material for: Mapping Geographic Trends in Early Childhood Social, Emotional, and Behavioural Difficulties in Glasgow: 2010–2017
Source: Int J Environ Res Public Health. 2022 Sep 13;19(18):11520. doi: 10.3390/ijerph191811520 (PMC9516987; doi:10.3390/ijerph191811520)
Supplement: Supplementary file 1 [file ijerph-19-11520-s001.zip › ijerph-1832013-supplementary.pdf]

Supplementary Table S1:

Preschool Demographics,

Years in study	Number of preschools	Children in preschools	Median SDQ IQR	% Most deprived	% Boys	% Outside expected age
**1**	16	125	5(2-9)	11 (8.8%)	61 (48.8%)	11 (8.8%)
**2**	6	262	5(2-9)	39 (14.9%)	148 (56.5%)	30 (11.4%)
**3**	7	340	4(1-9)	18 (5.29%)	165 (48.5%)	25 (7.4%)
**4**	21	1114	3(0-6)	122(11.3%)	528 (47.4%)	85 (7.6%)
**5**	24	2117	3(0-7)	406(19.2%)	1073 (50.7%)	130 (6.1%)
**6**	18	4342	5(2-9)	1209 (27.8%)	2255 (51.9%)	333 (7.7%)
**7**	28	7854	4(1-8)	1941 (24.7%)	4069 (51.8%)	604 (7.7%)
**8**	60	19 017	5(2-9)	5869 (30.9%)	9660 (50.8%)	1393 (7.3%)

Expected age of school start is 4.5-5.5. Deprivation measured using the most deprived quintile from Scottish Index of Multiple Deprivation.

Supplementary Table S2:

Demographics by Cohort

**Demographic**		**2010**	**2011**	**2012**	**2013**	**2014**	**2015**	**2016**	**2017**
**Age**	4-4.5	276 (9.0%)	281 (8.4%)	263 (6.8%)	198 (5.1%)	287 (5.4%)	256 (4.9%)	290 (5.3%)	261 (5.3%)
	4.5-5	133 (43.3%)	1574 (47.2%)	1816 (46.8%)	1802 (46.2%)	2527 (48.0%)	2492 (47.5%)	2572 (46.9%)	2340 (47.1%)
	5-5.5	1369 (44.4%)	1381 (41.4%)	1675 (43.1%)	1745 (44.8%)	2217 (42.0%)	2306 (44.0%)	2425 (44.3%)	2179 (44.8%)
	>5.5	104 (3.4%)	100 (3.0%)	128 (3.3%)	154 (4.0%)	244 (4.6%)	192 (3.7%)	193 (3.5%)	191 (3.8%)
**Sex**	F	1451 (47.1%)	1628 (48.8%)	1867 (48.1%)	1922 (49.3%)	2592 (49.1%)	2563 (48.4%)	2699 (49.3%)	2488 (50.1%)
	M	1631 (52.9%)	1708 (51.2%)	2015 (51.9%)	1977 (50.7%)	2683 (50.9%)	2683 (51.1%)	2781 (50.7%)	2483 (49.9%)
**SIMD Quintile**	5 (least)	515 (16.7%)	327 (9.8%)	507 (13.1%)	509 (13.1%)	891 (16.8%)	829 (15.8%)	827 (15.1%)	666 (13.4%)
	4	542 (17.6%)	493 (14.8%)	645 (16.6%)	585 (15.0%)	854 (16.2%)	852 (16.2%)	828 (15.1%)	811 (16.3%)
	3	566 (18.4%)	628 (18.8%)	674 (17.4%)	698 (17.9%)	1041 (19.7%)	1035 (19.7%)	1043 (19.0%)	1011 (20.3%)
	2	644 (20.9%)	808 (24.2%)	883 (22.7%)	966 (24.8%)	1162 (22.0%)	1200 (22.8%)	1341 (24.5%)	1175 (23.6%)

	1 (most)	815 (26.4%)	1080 (32.4%)	1173 (30.2%)	1141 (29.3%)	1327 (25.2%)	1330 (25.4%)	1441 (25.3%)	1308 (25.3%)

SIMD is Scottish Index of Multiple Deprivation.

Supplementary Table S3:

Demographics by Ward

	Ward	Population	% Boys	% Outside expected age	% Most deprived	% 2nd most deprived	% Middle deprived	% 2nd least deprived	% Least deprived
**1**	Anderston/City	860	449 (52.2%)	67 (7.8%)	27 (3.1%)	86 (10%)	191 (22.2%)	332 (38.6%)	224 (26%)
**2**	Baillieston	1912	988 (51.7%)	139 (7.3%)	487 (25.5%)	496 (25.9%)	289 (15.1%)	257 (13.4%)	383 (20%)
**3**	Calton	1374	731 (53.2%)	87 (6.3%)	862 (62.7%)	321 (23.4%)	176 (12.8%)	7 (0.5%)	8 (0.6%)
**4**	Canal	1854	921 (49.7%)	143 (7.7%)	896 (48.3%)	508 (27.4%)	239 (12.9%)	123 (6.6%)	88 (4.7%)
**5**	Craigton	1988	965 (48.5%)	164 (8.2%)	153 (7.7%)	531 (26.7%)	867 (43.6%)	398 (20%)	39 (2%)
**6**	Drumchapel/ Anniesland	2073	1061 (51.2%)	139 (6.7%)	1098 (53%)	317 (15.3%)	386 (18.6%)	235 (11.3%)	37 (1.8%)
**7**	East Centre	1906	972 (51%)	117 (6.1%)	732 (38.4%)	455 (23.9%)	341 (17.9%)	288 (15.1%)	90 (4.7%)
**8**	Garscadden/ Scotstounhill	1604	850 (53%)	111 (6.9%)	208 (13%)	574 (35.8%)	491 (30.6%)	165 (10.3%)	166 (10.3%)
**9**	Govan	1895	994 (52.5%)	125 (6.6%)	450 (23.7%)	806 (42.5%)	483 (25.5%)	148 (7.8%)	8 (0.4%)
**10**	Greater Pollok	2087	1076 (51.6%)	166 (8%)	565 (27.1%)	500 (24%)	265 (12.7%)	327 (15.7%)	430 (20.6%)
**11**	Hillhead	1052	515 (49%)	79 (7.5%)	92 (8.7%)	176 (16.7%)	140 (13.3%)	150 (14.3%)	494 (47%)
**12**	Langside	1183	582 (49.2%)	86 (7.3%)	0 (0%)	0 (0%)	129 (10.9%)	667 (56.4%)	387 (32.7%)

**13**	Linn	1821	906 (49.8%)	112 (6.2%)	608 (33.4%)	263 (14.4%)	235 (12.9%)	381 (20.9%)	334 (18.3%)
**14**	Maryhill/Kelvin	1828	947 (51.8%)	174 (9.5%)	466 (25.5%)	374 (20.5%)	357 (19.5%)	150 (8.2%)	481 (26.3%)
**15**	Newlands/Auldburn	1395	711 (51%)	118 (8.5%)	231 (16.6%)	191 (13.7%)	173 (12.4%)	310 (22.2%)	490 (35.1%)
**16**	North East	2485	1248 (50.2%)	213 (8.6%)	1058 (42.6%)	666 (26.8%)	212 (8.5%)	355 (14.3%)	194 (7.8%)
**17**	Partick West	1382	665 (48.1%)	86 (6.2%)	78 (5.6%)	190 (13.7%)	159 (11.5%)	288 (20.8%)	667 (48.3%)
**18**	Pollokshields	1378	696 (50.5%)	84 (6.1%)	65 (4.7%)	61 (4.4%)	209 (15.2%)	604 (43.8%)	439 (31.9%)
**19**	Shettleston	1689	872 (51.6%)	117 (6.9%)	564 (33.4%)	448 (26.5%)	438 (25.9%)	144 (8.5%)	95 (5.6%)
**20**	Southside Central	2077	1103 (53.1%)	208 (10%)	420 (20.2%)	724 (34.9%)	687 (33.1%)	246 (11.8%)	0 (0%)
**21**	Springburn	1328	709 (53.4%)	76 (5.7%)	555 (41.8%)	492 (37%)	229 (17.2%)	35 (2.6%)	17 (1.3%)

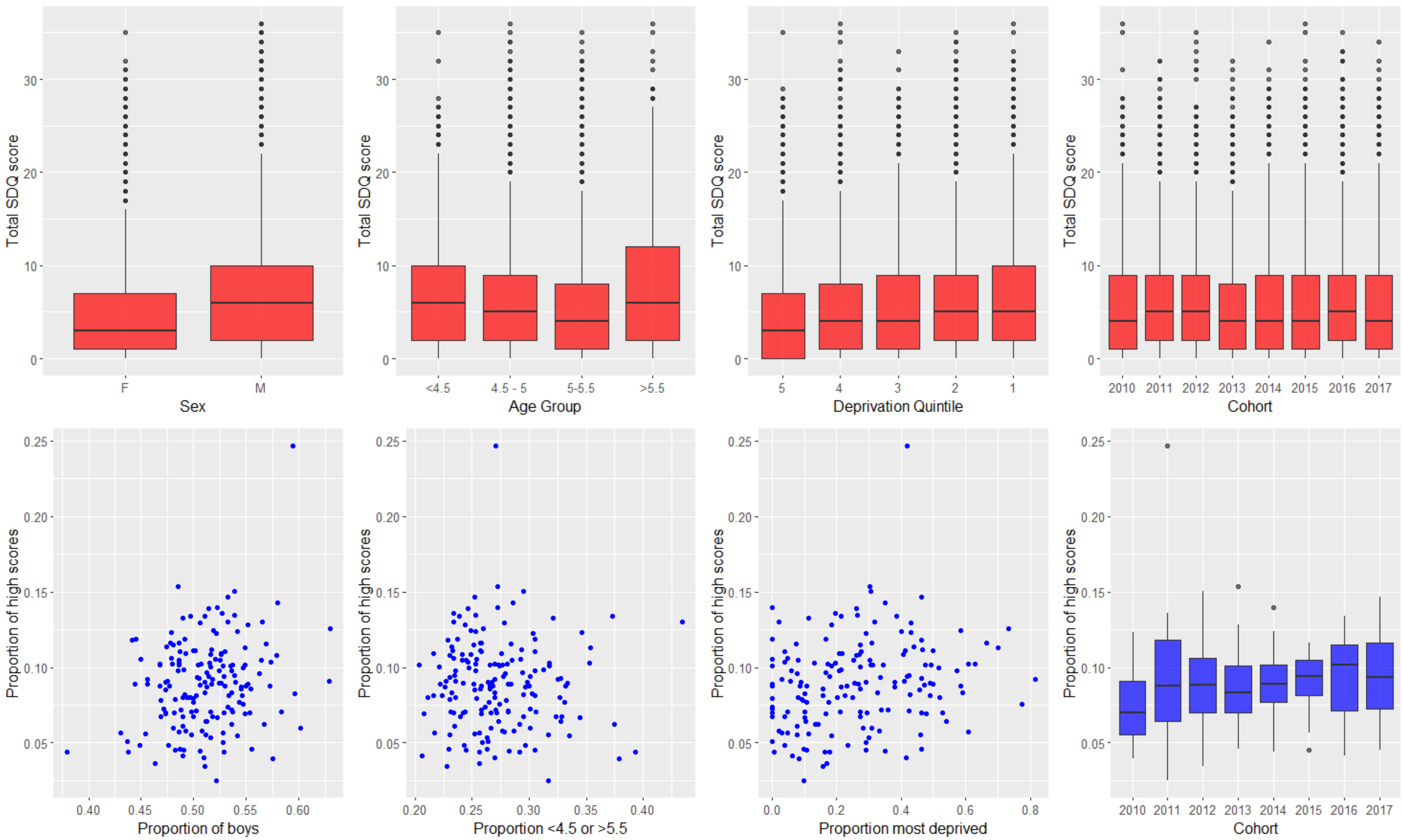

Supplementary Figure S1. **SDQ outcomes by covariates** Top panel shows total scores by sex, age group, deprivation quintile (5 =least deprived, 1 =most deprived) and cohort for all children (n=25,171). Bottom panel shows the proportion of children in each ward and year (n=168) with high SDQ scores by proportion of boys, children over 5 and most deprived and cohort.

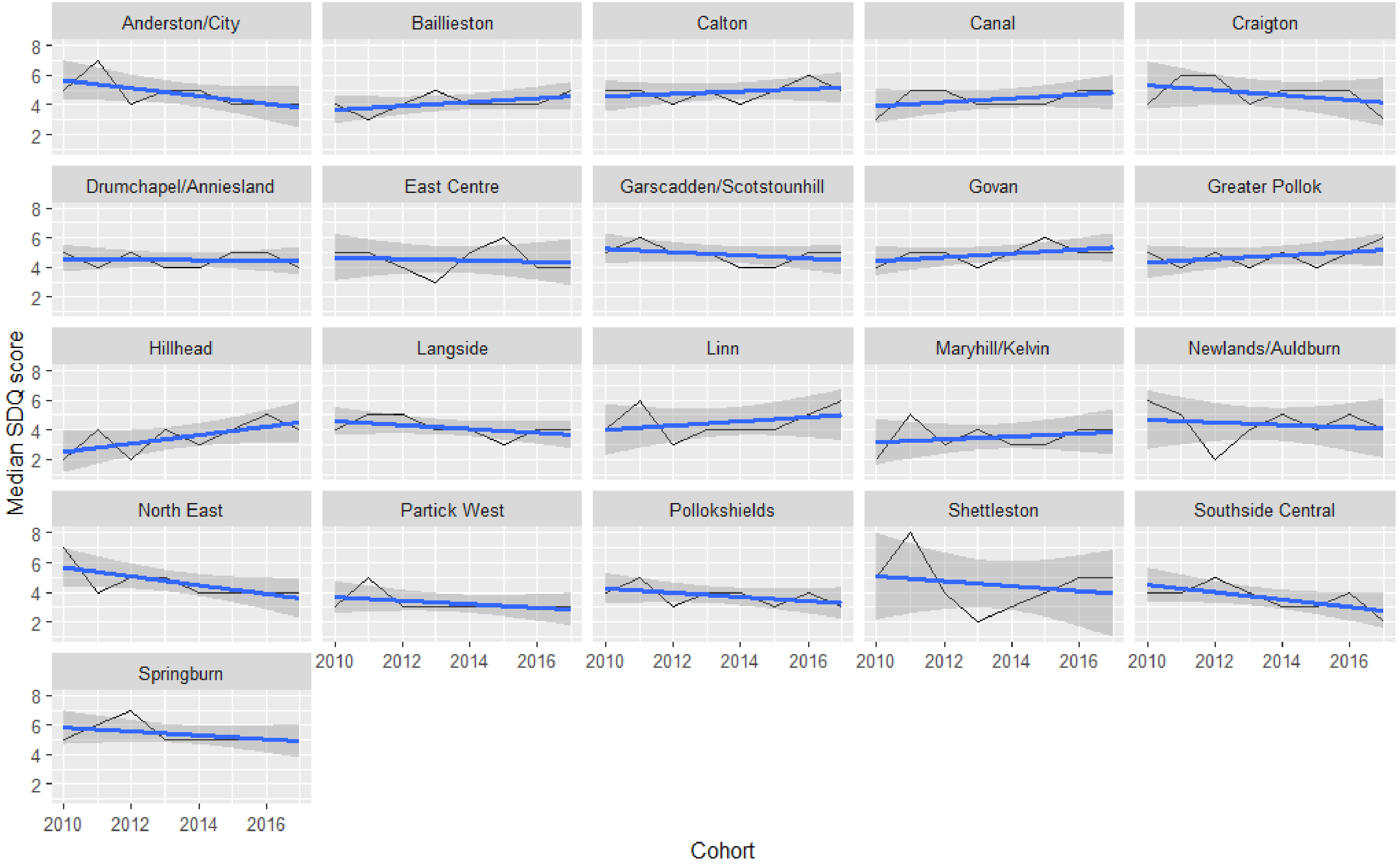

Supplementary Figure S2.

Median SDQ scores by Electoral Ward and Cohort

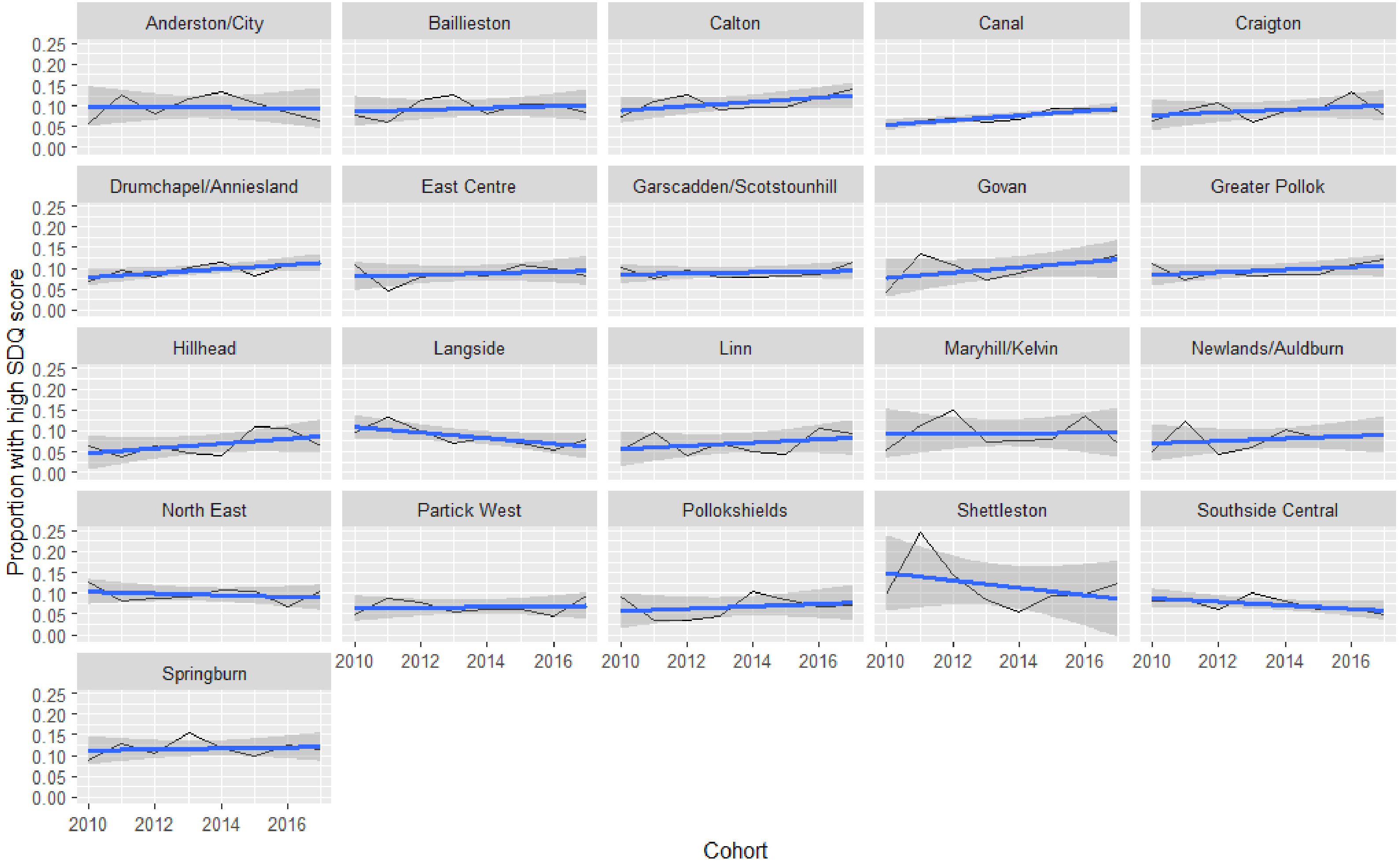

Supplementary Figure S3.

Proportion of children with high SDQ scores by Electoral Ward and Cohort
